# Modifications in steroid and triterpenoid metabolism in *Calendula officinalis* plants and hairy root culture in response to chitosan treatment

**DOI:** 10.1186/s12870-023-04261-4

**Published:** 2023-05-18

**Authors:** Agata Rogowska, Cezary Pączkowski, Anna Szakiel

**Affiliations:** Department of Plant Biochemistry, Faculty of Biology, University of Warsaw, 1 Miecznikowa Street, 02-096 Warsaw, Poland

**Keywords:** Chitosan, Elicitation, Sterols, Pentacyclic triterpenoids

## Abstract

**Background:**

Chitosan, a deacetylated derivative of chitin, is one of the most preferred biopolymers for use as biostimulants and biofertilizers in organic agriculture and as elicitors to enhance the productivity of plant in vitro cultures. Valued as a non-toxic, biodegradable, and environment-friendly agent, it is widely applied to improve plant growth and yield, the content of bioactive specialized metabolites, and resistance to stress conditions and pathogens. However, the influence of chitosan on the growth-defense trade-off, particularly the interplay between steroid and triterpenoid metabolism, has not been extensively investigated.

**Results:**

In this study, *Calendula officinalis* pot plants and hairy root cultures exposed to chitosan treatment displayed reduced biomass and altered steroid and triterpenoid metabolism. Biosynthesis and accumulation of free forms of sterols (particularly stigmasterol) were inhibited, while the content of sterol esters increased remarkably. The content of some triterpenoids (mainly free triterpenoid acids) was slightly enhanced; however, the biosynthesis of triterpenoid saponins was negatively affected.

**Conclusions:**

These results indicate that in certain plants, chitosan treatment might not positively influence the growth and metabolite production. Therefore, to avoid unexpected effects, initial studies of the conditions of chitosan treatment are recommended, including the dose and the number of chitosan applications, the type of treatment (e.g., foliar or soil), and the vegetative stage of the treated plants.

**Supplementary Information:**

The online version contains supplementary material available at 10.1186/s12870-023-04261-4.

## Introduction

Rising global food demand combined with dramatically changing environmental conditions resulting from climate change increase the need for agriculture adaptations and enhanced crop harvest worldwide. Due to often unpredictable field conditions, plants are exposed to a variety of stressors acting in tandem; therefore, holistic approaches are utilized to manage plant stress responses and to induce adaptation mechanisms. Out of concern for human health and the well-being of the habitat, new, less harmful solutions are sought to improve resistance to abiotic and biotic stresses and increase crop yields [1].

This search for eco-friendly, non-accumulative compounds that both promote plant growth and enhance crop productivity, referred to as biostimulants, is still underway. In addition to increasing plant yield and mineral uptake, biostimulants minimize the need to use chemical fertilizers, and biostimulant application has a positive effect on plant growth and disease prevention and reduces plant stress. Thus, it is not surprising that investigating new biostimulatory products shows increasing trends during the last decades [2].

Chitosan, a deacetylated derivative of chitin, is one of the seven main classes of biostimulants [3]. Chitin is a polysaccharide and the second most abundant natural polymer after cellulose. As a structural component of crustacean and insect exoskeletons, it is easily obtained from shellfish waste, but it also can be found in some fungal cell walls [4, 5]. Chitosan (β-1,4-linked glucosamine) is a more applicable form of chitin, as it can be easily modified without losing its innate properties [6], many of which make it an excellent candidate for agrotechnology applications, including being highly biodegradable, non-toxic and antimicrobial [7–10]. These characteristics suggest it may be an alternative to existing pathogen control methods that are primarily based on extensive use of agrochemicals and toxic pesticides. What is more, chitosan nanoparticles can be also used as nanocarriers of already existing agrochemicals like pesticides, fungicides, insecticides, and herbicides, enhancing their microbicide function and prolonging deliverance time [11].

Chitosan possesses eliciting properties that are manifested by the activation of numerous defense-related enzymes (i.e., ascorbate peroxidase, phenylalanine ammonia-lyase, catalase, and polyphenol oxidase), the accumulation of defense-related specialized metabolites (i.e., phytoalexins, phenolic compounds, callose, and lignin), the production of pathogenesis-related proteins (i.e., chitinase and β-1,3-glucanase), and the activation of a multi-leveled signal transduction network, which in turn enhances plant immunity [5, 12–15].

Chitosan has been determined to be an effective elicitor of specialized compounds, increasing their yield in numerous in vitro cultures and in pot plants as a foliar spray [16–19]. However, little is known about the effect of chitosan on general metabolites like sterols and the relation between sterols and specialized triterpenoids upon chitosan elicitation. As chitosan is a component of fungal cell walls, it was predicted that it might trigger defensive mechanisms connected with fungal pathogen attack, manifested by increased biosynthesis of specialized triterpenoids with anti-fungal properties or the rearrangement of the sterol profile to seal the cell membranes. Sterols and specialized triterpenoids (i.e., oleanolic acid) have a common chemical precursor – squalene [20] – and during stress conditions or elicitation, the biosynthesis pathways of these two groups may become competitive due to growth-defense trade-off, as has been previously reported [21–23].

The present study aimed to investigate the effect of chitosan on sterol and triterpenoid metabolism in two experimental models, *Calendula officinalis* hairy root cultures and greenhouse-cultivated plants (pot plants). Such an approach, applied previously for jasmonic acid and cadmium stress [22, 23], enables the comparison of the influence of a selected factor on an in vitro system and on the whole plant. The time points (for hairy root cultures: 3, 7, and 14 days; for pot plants: 7 and 14 days) were chosen to evaluate the response speed and biostimulant effect duration. In pot plants, the effect of chitosan foliar spray on chlorophyll content in leaves, as well as the contents of triterpenoids and carotenoids in inflorescences, were also evaluated.

## Results

### Chitosan effect on *C. officinalis* hairy root culture

Chitosan elicitation of a *C. officinalis* hairy root culture was performed as described in Materials and methods, section Chitosan elicitation of hairy roots. Chitosan concentration (50 mg/L) was selected based on previous reports [21] to allow a longer duration of the experiment and avoid roots decay. Control samples were treated with an equivalent volume of distilled water. The growth parameters and the contents of steroids and triterpenoids were measured at 3, 7, and 14 days after treatment.

### Effect of chitosan on hairy root biomass

**Fig. 1:**
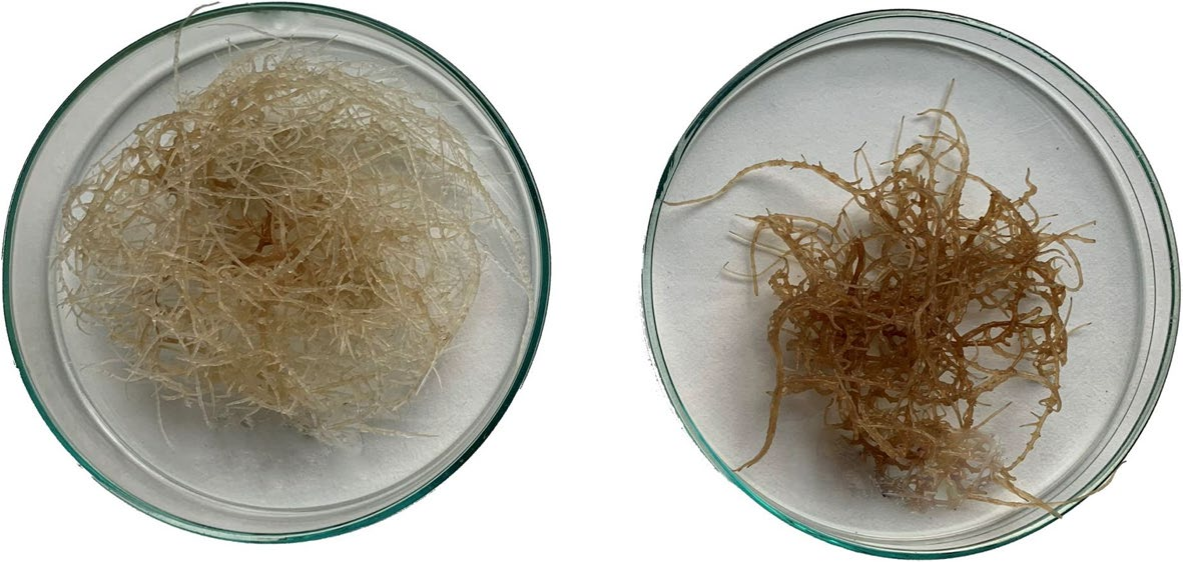
The morphological changes induced by chitosan in *C. officinalis* hairy roots after 3 days of treatment. Left- control, right- chitosan elicited sample

**Fig. 2:**
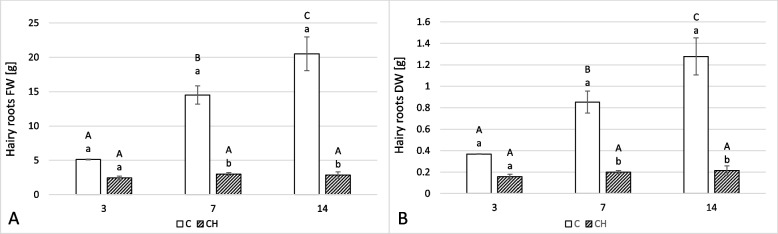
*C. officinalis* hairy root biomass FW (**A**) and DW (**B**) changes in time. C- control samples, CH- chitosan elicited samples. Bars which do not share a common letter are significantly different. Capital letters indicate significant difference in time between plants from the same treatment, lowercase indicate difference between treatments within certain time point

The presence of chitosan in the culture medium significantly altered the growth and appearance of *C. officinalis* hairy roots. After three days of incubation, the hairy root tissue was brown and less branched than the control samples (Fig. 1). The time-dependent effect of the applied elicitor on the hairy root biomass (expressed as fresh weight, FW, and dry weight, DW) is shown in Fig. 2. The fresh weight of elicited samples dropped by 52% (Fig. 2A) at 3 days, 79% by 7 days, and 86% by 14 days. Similarly, the dry weight of hairy roots dropped by 57%, 77%, and 83% after 3, 7, and 14 days, respectively (Fig. 2B).

### Chitosan influence on the content of steroids

GC–MS analysis of free steroid fraction from diethyl ether extracts of the control hairy roots confirmed the presence of the compounds previously described in Rogowska (2022) [22]. Among identified steroids, the main sterols were campesterol, cholesterol, isofucosterol, sitosterol, accompanied by its saturated form, sitostanol, and the predominating stigmasterol. Tremulone (stigmasta-3,5-dien-7-one) was the only steroid ketone found in the fraction. 24-methylene cycloartenol, a biosynthetic precursor of sterols, was also identified (Table S1).

**Fig. 3:**
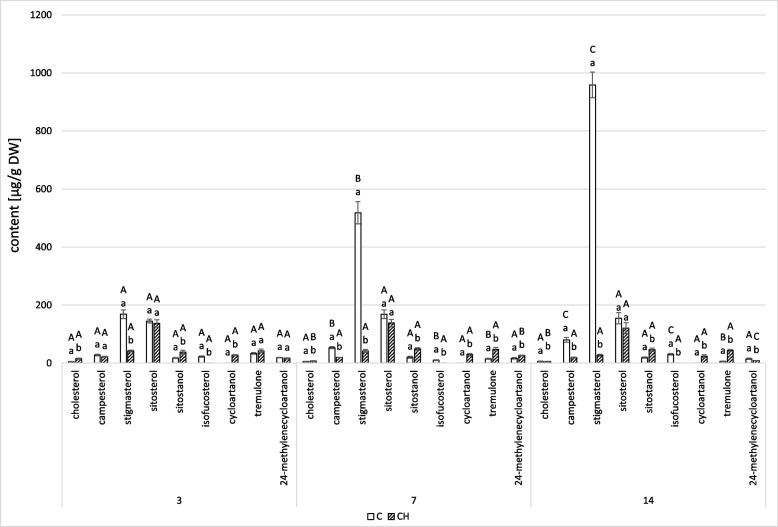
The effect of chitosan on steroid content. C- control samples, CH- chitosan elicited samples. Bars which do not share a common letter are significantly different. Capital letters indicate significant difference in time between plants from the same treatment, lowercase indicate difference between treatments within certain time point

Chitosan elicitation resulted in a significant decrease in the total steroid content in hairy roots (Fig. 3, Tables S2, S3). The total steroid content decreased by 22% at 3 days, by 55% at 7 days, and by 77% at 14 days, primarily the result of decreased stigmasterol, the most abundant steroid in the non-treated hairy roots. Consequently, sitosterol became the predominant steroid in chitosan-treated hairy roots, thereby affecting the stigmasterol to sitosterol ratio: 0.3:1 at 3 and 7 days, and 0.2:1 at 14 days. In contrast, the control samples showed an increased stigmasterol to sitosterol ratio: 1.2:1 at 3 days, 3.1:1 at 7 days, and 6.2:1 at 14 days.

Despite the remarkable decrease in total sterol content in elicited samples, elevated levels of some compounds, particularly cholesterol, sitostanol, and tremulone, were observed. This increase was most remarkable for cholesterol at 3 days, demonstrating a nearly three-fold increase. In comparison, sitostanol increased twofold over the 14 days, while the level of tremulone rose gradually to 27% at 3 days, then to over threefold at 7 days, and finally to more than sevenfold at 14 days. Notably, isofucosterol was absent in the sterol profile of samples treated with chitosan. Instead, cycloartanol, being the saturated form of biosynthetic precursor cycloartenol absent in the control samples, appeared in elicited hairy roots.

The ester and glycoside fractions of diethyl ether and methanol extracts subjected to hydrolysis consisted of four sterols, i.e., campesterol, cholesterol, stigmasterol, and sitosterol, as described in the previous reports [22, 23].

**Fig. 4:**
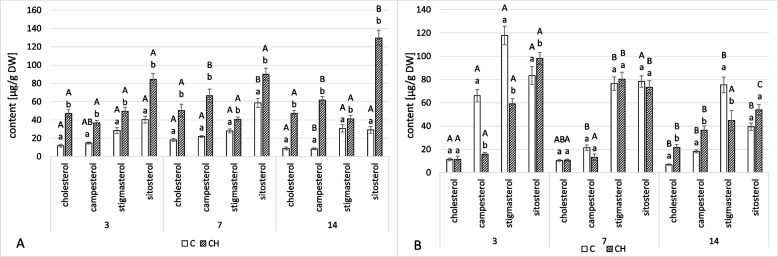
Content of sterol esters (**A**) and sterol glycosides (**B**) in *C. officinalis* hairy roots tissue. C- control samples, CH- chitosan elicited samples. Bars which do not share a common letter are significantly different. Capital letters indicate significant difference in time between plants from the same treatment, lowercase indicate difference between treatments within certain time point

The total content of sterol ester forms was significantly higher in chitosan-treated hairy roots than in non-treated samples throughout the whole experiment (Fig. 4A, Tables S4, S6). At 3 days, elicited samples showed a sterol ester content 129% higher than in control samples at the same time point; at 7 days, it was 96% higher; at 14, it was 264% higher. Sitosterol was the dominant compound in sterol ester forms in both control and elicited samples.

In contrast, the total content of sterol glycosides at first decreased in chitosan-treated hairy roots, by 34% and 5% after 3 and 7 days, respectively (Fig. 4B, Tables S5, S6). However, at 14 days, it was 12% higher in elicited samples compared to control. In non-treated hairy roots, stigmasterol was the dominant glycoside sterol compound (sitosterol and stigmasterol were at similar levels only after 7 days), while in elicited samples the dominant compound was mostly sitosterol (stigmasterol was the most abundant only once, after 7 days).

### Effect of chitosan elicitation on the content of triterpenoids

**Fig. 5:**
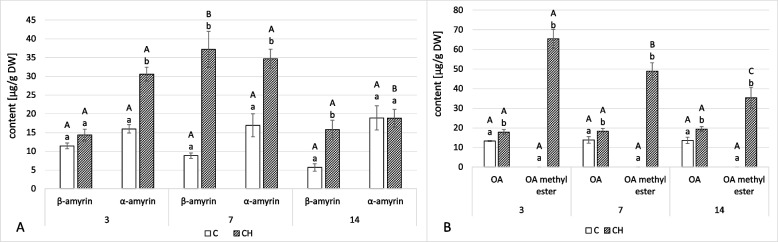
Content of neutral triterpenoids (amyrins) (**A**) and free oleanolic acid and its methyl ester (**B**) in hairy roots tissue. C- control, CH- chitosan elicited samples. Bars which do not share a common letter are significantly different. Capital letters indicate significant difference in time between plants from the same treatment, lowercase indicate difference between treatments within certain time point

As in previous reports, two triterpenoid alcohols were found in diethyl ether extracts from hairy roots: α-amyrin, the precursor of triterpenoids, distinguished by an ursane-type skeleton, and β-amyrin, the precursor of oleanane-type triterpenoids. α-amyrin was the dominant compound in non-treated hairy roots (Fig. 5A, Tables S7, S8).

Chitosan elicitation caused an increased biosynthesis of both alcohols. After 3 days, the level of α- and β-amyrin was 92% and 25%, respectively, higher in elicited roots than in controls. The most remarkable change was noted at 7 days when α-amyrin concentration was over twofold, and β-amyrin over fourfold, compared to the controls, resulting in β-amyrin content slightly higher than α-amyrin. At 14 days, the β-amyrin content in elicited samples was almost threefold higher compared to non-treated roots, though it was more than twice lower than in samples collected after 7 days of treatment. The level of α-amyrin in elicited samples collected after 14 days of treatment was nearly the same as in the control samples; however, as in the case of β-amyrin, the actual amount present in the sample had decreased by almost twice lower over the 7 days.

The elevated content of β-amyrin was coupled with the increased biosynthesis of free oleanolic acid (OA) and its endogenous methyl ester found in hairy root tissue (Fig. 5B, Tables S9, S10). Interestingly, the latter was not detected in the control roots. After 3 and 7 days, the content of free oleanolic acid increased by 35% and 32%, respectively, and after 14 days its level was 43% higher than in control roots. Oleanolic acid methyl ester content was the highest after 3 days, reaching 65 μg/g of dried weight, and it gradually decreased throughout the rest of the experiment.

**Fig. 6:**
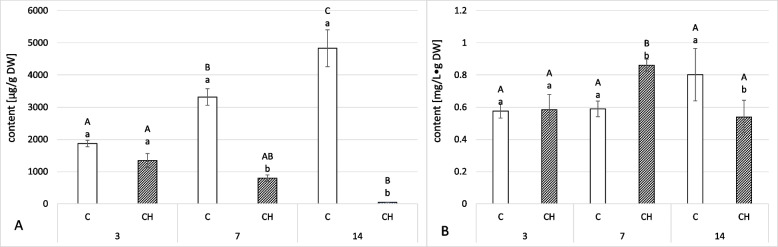
Content of saponins in the root tissue (**A**) and released to the culture medium (**B**). C- control, CH- chitosan elicited samples. Bars which do not share a common letter are significantly different. Capital letters indicate significant difference in time between plants from the same treatment, lowercase indicate difference between treatments within certain time point

Elicitation with chitosan had the opposite effect on the concentration of oleanolic acid saponins in hairy root tissue (Fig. 6A, Tables S11, S12). Saponins in elicited hairy roots gradually decreased throughout the experiment, while in the control samples it gradually increased: at 3 days, elicited samples had saponin content 28% lower than controls, but the concentration dropped substantially after that, to 76% and 99% at 7 and 14 days, respectively.

In contrast, the saponins in the elicited and control culture medium showed less substantial differences (Fig. 6B, Tables S13, S14). At 3 days, saponin content was nearly identical in control and elicited samples; at 7 days, saponin content was 46% higher in chitosan-elicited samples; at 14 days, it was 33% lower.

### Effect of chitosan elicitation on *C. officinalis* plants

Three-week-old *C. officinalis* plants growing in the greenhouse were sprayed with a 50 mg/L solution of chitosan, as described in Materials and methods, Section Chitosan elicitation of pot-cultures. Control plants were sprayed with an equivalent amount of water. Growth parameters, defined as the concentrations of steroids and triterpenoids, were measured separately in roots and aerial parts (shoots) at 7 and 14 days of cultivation. Additionally, at 7 and 14 days of post-treatment cultivation, the chlorophyll in the leaves and the carotenoids content in inflorescences were measured.

### Chitosan effect on root and shoot growth parameters

**Fig. 7:**
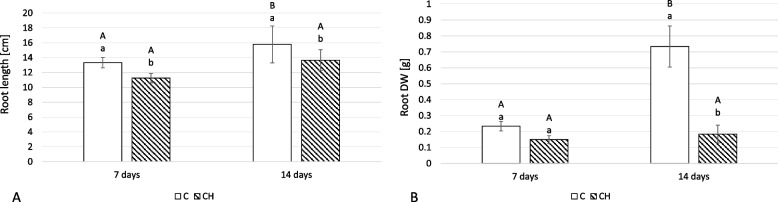
The length (**A**) and dry weight DW (**B**) of *C. officinalis* plant roots. C- control, CH- chitosan elicited samples. Bars which do not share a common letter are significantly different. Capital letters indicate significant difference in time between plants from the same treatment, lowercase indicate difference between treatments within certain time point

Chitosan treatment negatively impacted *C. officinalis* plant growth, as manifested by a decrease in length and biomass (dried weight) of roots and shoots. At 7 days of post-treatment cultivation, the root length of treated plants was shorter by 16% in comparison to control roots at 14 days, they were 14% shorter (Fig. 7A). Dried weight also decreased, though to a greater degree: at 7 days, it was 36% less; at 14 days, it was 75% less (Fig. 7B).

**Fig. 8:**
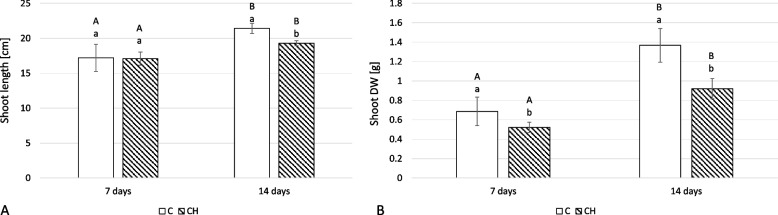
The length (**A**) and dry weight DW (**B**) of *C. officinalis* plant shoots. C- control, CH- chitosan elicited samples. Bars which do not share a common letter are significantly different. Capital letters indicate significant difference in time between plants from the same treatment, lowercase indicate difference between treatments within certain time point

In shoots, as in roots, a stronger effect was detected on biomass (dried weight) compared to length. At 7 days, there was no significant difference in shoot lengths (Fig. 8A); at 14 days, the shoot length of elicited plants decreased by 10% (Fig. 8A). In comparison, at 7 days, the dried weight of elicited samples was 24% lower; at 14 days, it was 33% lower (Fig. 8B).

### Chitosan influence on steroid content in *C. officinalis* roots

As reported in Rogowska 2022 [22, 23], *C. officinalis* roots were composed of cholesterol, campesterol, predominant stigmasterol, sitosterol, as well as its fully hydrogenated derivative, sitostanol; three steroidal ketones: tremulone, sitostenone, and stigmastan-3,6-dione; and one derivative of the sterol precursor cycloartenol acetate. In contrast to the steroid profile of the hairy roots, neither isofucosterol nor 24-methylene cycloartenol was detected.

**Fig. 9:**
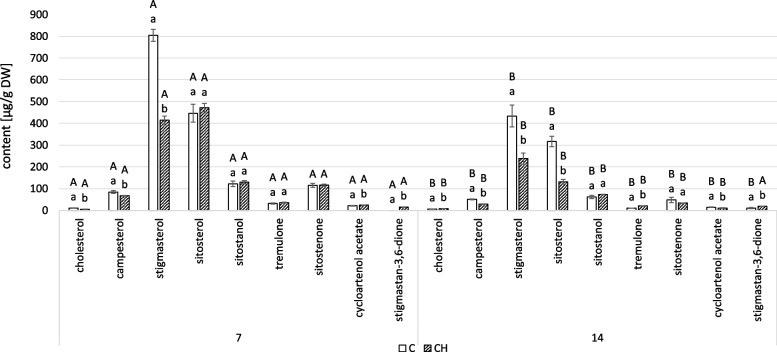
Content of steroids in *C. officinalis* roots. C- control, CH- chitosan elicited samples. Bars which do not share a common letter are significantly different. Capital letters indicate significant difference in time between plants from the same treatment, lowercase indicate difference between treatments within certain time point

Chitosan treatment had an inhibitory effect on steroid biosynthesis and accumulation in *C. officinalis* roots, similar to the hairy root cultures. At 7 and 14 days after elicitation, the total steroid content in the roots of the elicited plants was 22% and 41%, respectively, that of non-treated samples (Fig. 9, Tables S15, S16).

Chitosan also affected the ratio of stigmasterol to sitosterol. As in hairy roots, at 7 days, sitosterol was the dominant compound in elicited samples: the stigmasterol to sitosterol ratio in treated samples was 0.9:1, while in controls it was 1.8:1. However, at 14 days after treatment, stigmasterol was again the dominant compound in both elicited and control samples, and samples had a stigmasterol to sitosterol ratio of 1.8:1 and 1.4:1, respectively.

**Fig. 10:**
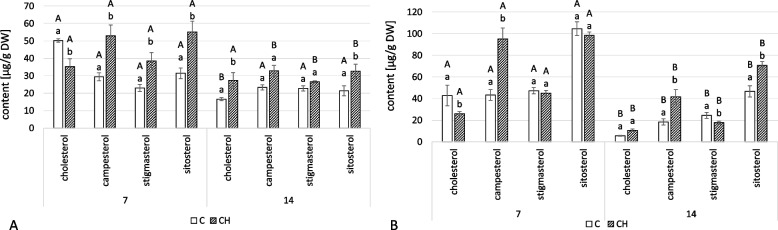
Content of sterol esters (**A**) and sterol glycosides (**B**) in *C. officinalis* roots. C- control, CH- chitosan elicited samples. Bars which do not share a common letter are significantly different. Capital letters indicate significant difference in time between plants from the same treatment, lowercase indicate difference between treatments within certain time point

As in the hairy roots, four sterols – cholesterol, campesterol, stigmasterol, and sitosterol – were found in conjugated forms, namely sterol esters and sterol glycosides (Fig. 10, Tables S19, S20). In the roots of chitosan-treated plants, the total content of sterols in ester forms was higher by 36% and 42% at 7 and 14 days, respectively (Fig. 10A). The proportions between individual compounds also differed in control and elicited samples. At 7 days, the dominant sterol in ester forms was cholesterol in control plants but sitosterol in elicited ones. At 14 days, the dominant compound in esterified sterols was campesterol in both samples.

Chitosan elicitation also increased the total concentration of sterol glycosides in the roots of treated plants, showing concentrations that were 11% and 48% higher at 7 and 14 days, respectively (Fig. 10B). Sitosterol was the dominant compound throughout the experiment in both elicited samples and controls, though campesterol more than doubled in elicited samples at both time points.

### Chitosan effect on the content of steroids in *C. officinalis* shoots

**Fig. 11:**
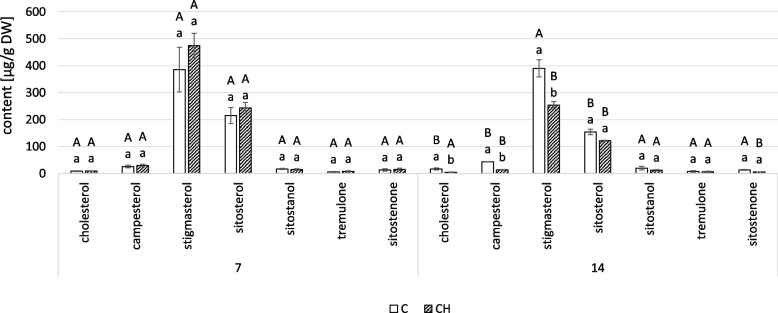
Content of steroids in *C. officinalis* shoots. C- control, CH- chitosan elicited samples. Bars which do not share a common letter are significantly different. Capital letters indicate significant difference in time between plants from the same treatment, lowercase indicate difference between treatments within certain time point

The profile of steroids present in free forms in *C. officinalis* shoots consisted of cholesterol, campesterol, stigmasterol, sitosterol, and sitostanol, as well as two ketones, tremulone, and sitostenone [22, 23]. At 7 days, the content of steroids in shoots of chitosan-elicited plants was 18% higher than in controls, while at 14 days it was 35% lower (Fig. 11, Tables S21, S22), with all identified compounds lower than in controls. The dominant compound throughout the whole experiment in both control and elicited samples was stigmasterol, followed by sitosterol. The 7 and 14-day stigmasterol to sitosterol ratios was 1.8:1 and 2.5:1 in controls and 1.9:1 and 2.1:1 in elicited samples.

**Fig. 12:**
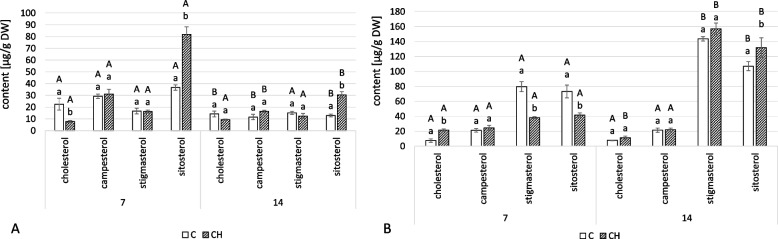
Content of sterol esters (**A**) and sterol glycosides (**B**) in *C. officinalis* shoots. C- control, CH- chitosan elicited samples. Bars which do not share a common letter are significantly different. Capital letters indicate significant difference in time between plants from the same treatment, lowercase indicate difference between treatments within certain time point

The total content of sterols in ester forms was higher in elicited plants than in controls, by 30% and 27% at 7 and 14 days, respectively (Fig. 12A, Tables S25, S26). The greatest increase was in the sitosterol esters, which was more than twice as high in the samples collected both at 7 and 14 days after treatment. In contrast, the concentration of cholesterol esters slightly decreased, whereas campesterol and stigmasterol remained at similar levels.

The total content of sterols in glycoside forms was also affected by the chitosan treatment (Fig. 12B, Tables S25, S26). At 7 days, the content of sterol glycosides was 31% less than the control, but at 14 days, it was 15% higher. The predominant compounds differed between the treatment and control samples: while stigmasterol was the dominant compound in the control plants at both time points, the elicited samples had different compounds dominant at the two samplings – sitosterol was dominant at 7 days while stigmasterol was dominant at 14.

### Chitosan effect on the content of triterpenoids in *C. officinalis* roots and shoots

**Fig. 13:**
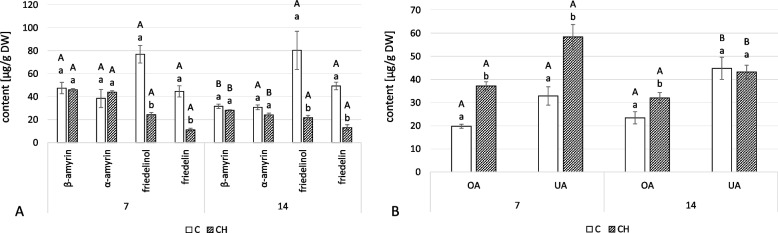
Content of neutral triterpenoids (**A**) and free acids (**B**) in *C. officinalis* roots. C- control, CH- chitosan elicited samples. Bars which do not share a common letter are significantly different. Capital letters indicate significant difference in time between plants from the same treatment, lowercase indicate difference between treatments within certain time point

The neutral triterpenoids profile of marigold roots comprised three triterpenoid alcohols, α-amyrin, β-amyrin and friedelinol, and one ketone, friedelin [22, 23]. Chitosan elicitation decreased the total content of neutral triterpenoids in *C. officinalis* roots; at 7 and 14 days, they were 40% and 55% percent lower than controls, respectively (Fig. 13A, Table S17). The sharpest decline was in the most abundant compound, friedelinol, which decreased by more than threefold, and ketone friedelin (approximately fourfold) in elicited samples. The contents of both amyrins remained at similar levels to controls and showed no substantial difference.

Oleanolic acid and its isomer, ursolic acid, were identified in the fraction of free acids from *C. officinalis* roots. At 7 days of the experiment, the content of both acids was higher in the elicited samples than the controls: 88% for oleanolic acid and 78% for ursolic acid (Fig. 13B, Tables S15, S18). At 14 days, differences between elicited and control samples were less substantial: oleanolic acid was 37% higher, and ursolic acid was 4% lower.

**Fig. 14:**
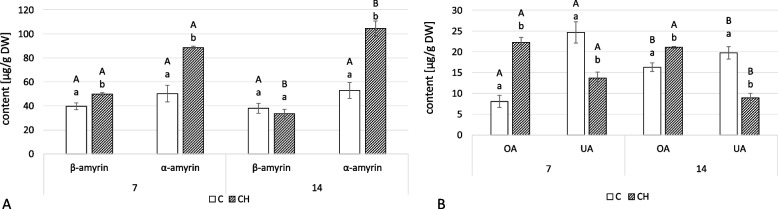
Content of neutral triterpenoids (**A**) and free acids (**B**) in *C. officinalis* shoots. C- control, CH- chitosan elicited samples. Bars which do not share a common letter are significantly different. Capital letters indicate significant difference in time between plants from the same treatment, lowercase indicate difference between treatments within certain time point

In the fraction of neutral triterpenoids from *C. officinalis* shoots, only two amyrins were found: α- and β-amyrin (Fig. 14A, Tables S21, S23). At 7 days of the experiment, the content of both amyrins was higher in the elicited samples: β-amyrin by 26%, α-amyrin by 76%. At 14 days, the content of β-amyrin was slightly less (12%), whereas α-amyrin content was substantially higher (almost two-times).

Chitosan elicitation substantially affected the proportion of the two isomers, oleanolic and ursolic acid, that occur in free form in *C. officinalis* shoots (Fig. 14B, Tables S21, S24). Ursolic acid was dominant in the control plants throughout (though its content slightly decreased at 14 days), whereas oleanolic acid was dominant in the elicited samples. The differences between elicited and control plants were particularly visible at 7 days of the experiment: in elicited samples compared to controls, oleanolic acid concentration was 2.7 times higher, but ursolic acid concentration was 44% lower.

**Fig. 15:**
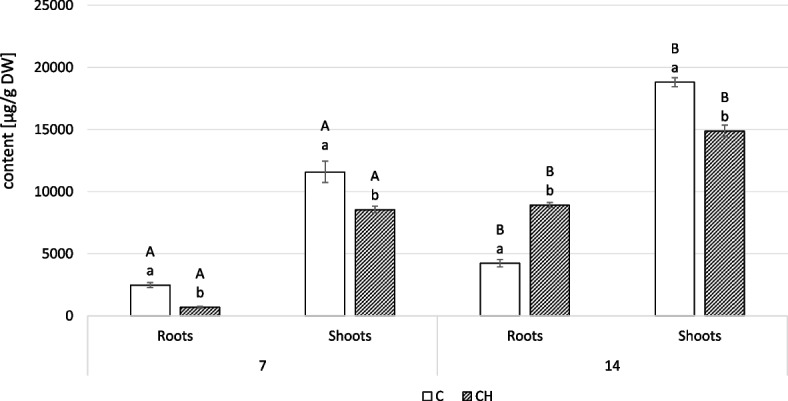
Content of saponins in roots and shoots of *C. officinalis* plants. C- control, CH- chitosan elicited samples. Bars which do not share a common letter are significantly different. Capital letters indicate significant difference in time between plants from the same treatment, lowercase indicate difference between treatments within certain time point

Chitosan elicitation also affected the saponin content in both roots and shoots of *C. officinalis* plants (Fig. 15, Tables S27, S28). At 7 days after elicitation, root saponin content was 3.5-fold lower in elicited plants compared to controls; however, at 14 days, this trend was reversed: saponin content in elicited plants was more than double that of controls. The changes in saponin content observed in shoots were more stable, lower by 26% and 21% at 7 and 14 days, respectively.

### Chitosan effect on the content of steroids and triterpenoids in *C. officinalis* inflorescences

**Fig. 16:**
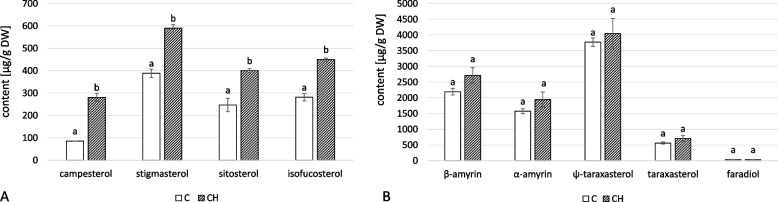
Content of free sterols (**A**) and neutral triterpenoids (**B**) in *C. officinalis* inflorescences. C- control, CH- chitosan elicited samples. Bars which do not share a common letter are significantly different

Inflorescences contained four free sterols: campesterol, stigmasterol, sitosterol, and isofucosterol. Chitosan elicitation substantially increased the contents of all sterols, resulting in a total control-to-treatment increase of 72% (Fig. 16A, Tables S29, S30). The dominant compound in both elicited and control samples was stigmasterol. In addition to α- and β- amyrin, three neutral triterpenoid alcohols were identified: ψ-taraxasterol, taraxasterol, and dihydroxy alcohol faradiol, all of which were found exclusively in this organ of *C. officinalis*. Chitosan elicitation was associated with a slightly higher concentration of total neutral triterpenoids (16%); however, the differences between control and elicited samples were not statistically significant (Fig. 16B, S29, S31). The dominant compound in this fraction for both samples was ψ-taraxasterol.

**Fig. 17:**
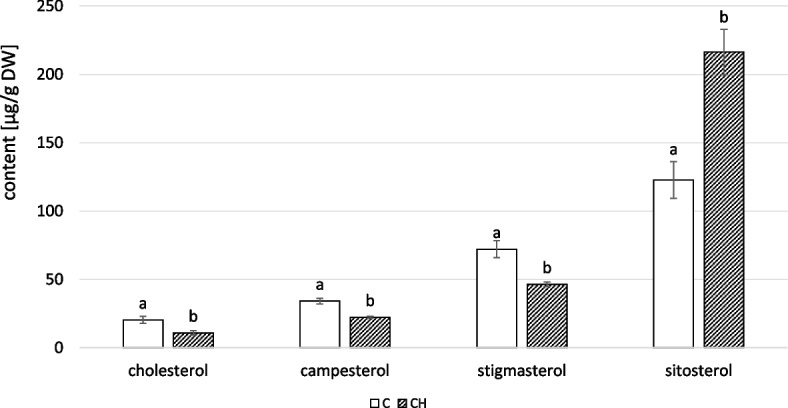
Content of sterol glycosides in *C. officinalis* inflorescences. C- control, CH- chitosan elicited samples. Bars which do not share a common letter are significantly different

Among the sterol glycosides in *C. officinalis* inflorescences, cholesterol, campesterol, stigmasterol, and sitosterol were identified. While sitosterol was dominant in control and elicited samples, it was substantially higher in elicited samples, by 76%, whereas the remaining sterols decreased (Fig. 17, Tables S33, S34).

**Fig. 18:**
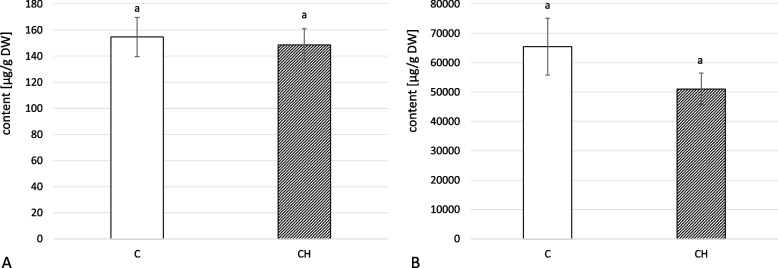
Content of free oleanolic acid (**A**) and oleanolic acid saponins (**B**) in *C. officinalis* inflorescences. C- control, CH- chitosan elicited samples. Bars which do not share a common letter are significantly different

The inflorescences of *C. officinalis* with chitosan elicitation did show a decrease in saponin content (22%); however, neither this change (Fig. 18B, Tables S29, S32, S35, S36) nor the changes in free oleanolic acid (Fig. 18A) were statistically significant.

### Chitosan effect on the chlorophyll content in leaves of *C. officinalis*

**Fig. 19:**
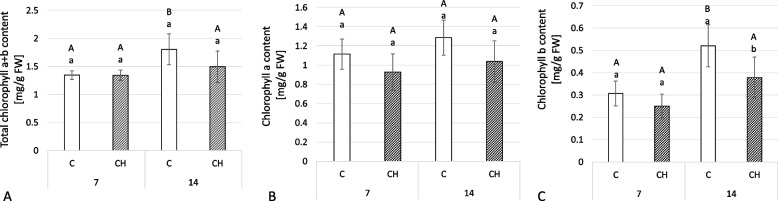
Content of total chlorophyll a + b (**A**), chlorophyll a (**B**) and chlorophyll b (**C**) in *C. officinalis* leaves. C- control, CH- chitosan elicited samples. Bars which do not share a common letter are significantly different. Capital letters indicate significant difference in time between plants from the same treatment, lowercase indicate difference between treatments within certain time point

While samples from chitosan-elicited plants showed a decreased chlorophyll content compared to controls (Fig. 19), the only statistically significant difference was a 27% decrease in chlorophyll b content (Fig. 19C).

### Chitosan effect on the content of carotenoids in *C. officinalis* inflorescences

**Fig. 20:**
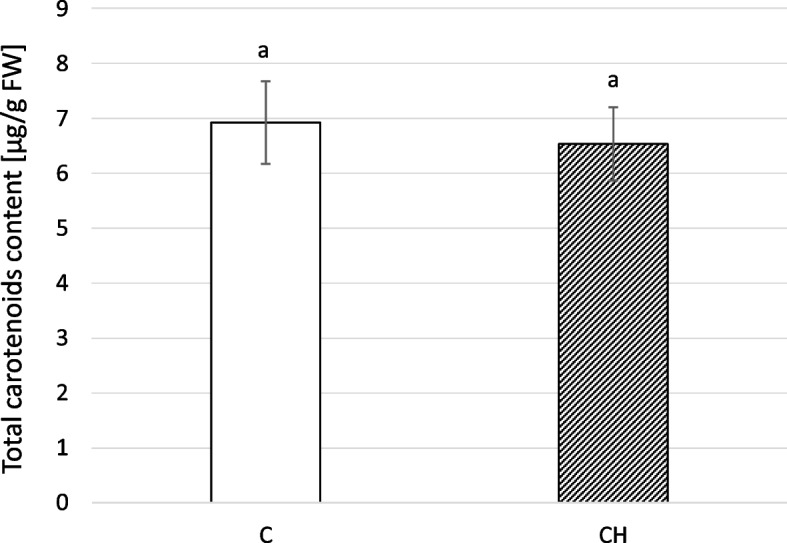
Total carotenoids content in *C. officinalis* inflorescences. C- control, CH- chitosan elicited samples. Bars which do not share a common letter are significantly different

As in the case of total chlorophyll content in leaves, chitosan treatment had no significant influence on total carotenoid content in *C. officinalis* inflorescences (Fig. 20).

## Discussion

The present study aimed to evaluate the effect of chitosan on plant growth as well as on general and specialized metabolism, represented by steroids and triterpenoids, respectively, in *C. officinalis* plants and hairy root cultures. General metabolites like sterols play a major role in plant growth and development, while specialized metabolites like pentacyclic triterpenoids are regarded as defensive compounds. Chitosan, as a potential stimulator of plant responses to pathogen attacks, may have a significant influence on changes in the profiles of those two groups of metabolites.

In *C. officinalis* hairy root cultures, chitosan elicitation caused a dramatic growth inhibition, manifested by a decreased biomass (up to 86%), morphological changes, and visible tissue browning, results that confirmed previous observations [21]. Chitosan, applied as an elicitor, exerted growth inhibition, as has been found in studies of in vitro cultures of other species, including *Withania somnifera* [24], *Hypericum perforatum* [25], *Morinda coreia* [26], and *Dysphania ambrosioides* [27]; however, compared to our study, studies on these models indicated a less substantial negative effect on biomass.

Growth inhibition was also observed after chitosan treatment of *C. officinalis* pot plants, though to a lesser extent for hairy root culture. This was primarily manifested by a decrease in dried weight (up to 33% for shoots, and 75% for roots), indicating that root growth was more substantially affected than the directly treated shoots and the presence of efficient signal transduction to the roots after the foliar application of chitosan. In contrast to these results, Abdel-Wahed (2020) [28] found a positive effect on *C. officinalis* plant growth after chitosan treatment for powdery mildew (*Erysiphe cichoracearum*) infection. However, the conditions of these experiments differed substantially, with Abdel-Wahed (2020) utilizing a longer duration with more numerous treatments (two foliar applications repeated every two weeks). This suggests that these factors have a substantial impact on the ultimate effect of chitosan on plant growth parameters.

Chitosan elicitation negatively affected the steroid content in both hairy root cultures (77% less) and *C. officinalis* pot plant roots (41% less). This effect was less evident in the shoots, which first increased slightly and then decreased (17% increase and 35% decrease). A decrease in sterol biosynthesis would be expected to cause a biomass reduction since sterols are indispensable constituents of cellular membranes. The decrease in stigmasterol content in *C. officinalis* hairy roots and roots upon chitosan elicitation is particularly spectacular (35-fold in hairy roots; twofold in the roots of the treated plant), which is unsurprising given its high concentration among free sterols in untreated samples. Stigmasterol is one of the principal sterols responsible for building plant cell plasma membranes; thus, fluctuations in its content as well as changes in the ratio between stigmasterol and other sterols (i.e., sitosterol) are often reported as a key characteristic indicator of stress [29]. For example, a decrease in stigmasterol content was reported in roots of *Cucumis sativus* (cucumber), *Glycine max* (soybean), *Solanum lycopersicum* (tomato), and *Zea mays* (corn) after infection by plant parasitic nematode, *Meloidogyne incognita* [30]. Tomato plants infected by *M. incognita* were characterized by decreased levels of stigmasterol and the repression of the gene CYP710A11, encoding the sterol C-22 desaturase, which is responsible for the conversion of β-sitosterol to stigmasterol [31]. Similar results were observed after cotton infection with the fungal pathogen, *Verticillium dahliae*, causing wilt disease: the content of total sterol and as well as each type of sterol, including stigmasterol, decreased in infected cotton roots. Furthermore, in transgenic cotton plants overexpressing the sterol C22-desaturase gene, and thus characterized by a higher content of stigmasterol, the resistance to Verticillium wilt significantly increased [32]. On the other hand, elevated content of stigmasterol was detected in *Arabidopsis* leaves after inoculation with *Pseudomonas syringae*, a highly dangerous bacterial plant pathogen [33]. These results suggest that stigmasterol and sitosterol are important compounds in plant–microbe interactions. However, defense responses involving modifications in sterol metabolism are not uniform. In the present study, sterol metabolism was particularly affected after chitosan treatment of hairy roots, where other features pointed to the rearrangement of sterol biosynthetic pathways (e.g., the absence of isofucosterol, the increased content of cholesterol and steroid ketone tremulone). The accumulation of cycloartanol, the saturated form of sterol precursor cycloartenol, suggests the partial inhibition of the downstream steps of biosynthesis.

Chitosan elicitation also changed the content of conjugated forms of sterols. In hairy roots, as well as in roots and shoots of elicited plants, the content of sterol esters increased during the experiment (Figs. 4A, 10A, and 12A). The conversion of sterols into sterol esters, thereby ensuring a steady supply of membrane sterols, is one mechanism for the maintenance of plasma membrane integrity in response to stress in plants [34]. In both experimental models, hairy roots and pot plants of *C. officinalis*, there was a decrease in the content of free sterols as well as an increase in sterol esters; sterol turnover via esterification reaction seems to be an important strategy in plant response to elicitors like chitosan. In previous studies on cadmium ions and jasmonic acid, sterol esters were also affected, though not uniformly [22, 23]. Changes in the content of sterol glycosides upon chitosan elicitation were more variable; in hairy root cultures and shoots, the initial decrease in sterol glycosides was followed by an increase, though one only found in roots (Figs. 4B, 10B, and 12B). The content of sterol glycosides can be affected by various stress factors like cold, drought, and salt stress [35–37]; increased sterol glycosylation was observed after treatment with jasmonic acid of both hairy roots and *C. officinalis* plants in the previous study [23].

To date, many studies have utilized chitosan as an elicitor, which has been shown to effectively enhance the biosynthesis of pharmaceutically useful compounds in both in vitro and *in planta* systems. In hairy root cultures, chitosan elicitation has increased the content of specialized compounds such as flavonoids, triterpenoid saponins, and terpenoid alkaloids [38–40]. However, the present study found no such stimulation – the content of saponins in hairy root tissue decreased in time, and the content of saponins released to the culture medium first increased and then decreased, showing a non-uniform pattern (Fig. 6). Free oleanolic acid content remained elevated in elicited samples throughout (Fig. 5B). Particularly interesting was the presence of oleanolic acid methyl ester in elicited samples. None of the previous studies reported this compound under elicitation or stress factor treatment; oleanolic acid was only present in a free form or as saponins. Nevertheless, it can be concluded that in the case of *C. officinalis* hairy root cultures, chitosan was not as efficient an elicitor of specialized metabolites as, for example, jasmonic acid, whose ability to enhance biosynthesis and secretion of oleanolic acid saponins has been demonstrated in previous studies [21, 23].

Chitosan treatment has also been used in several studies to increase the biosynthesis of specialized metabolites *in planta*. For example, a foliar spray with chitosan lactate increased the content of rosmarinic acid and anthocyanins in lemon balm (*Melissa officinalis*) [41]. Chitosan application on the grapevine (*Vitis vinifera* L. cv. Tinto Cão) has positively affected the accumulation of anthocyanins and other phenolics in fruit skins [42]. In the present study, increased oleanolic and ursolic acids were detected in roots of *C. offcinalis* after 7 days of elicitation, though this difference diminished after 14 days, leaving only oleanolic acid elevated (Fig. 13B). In shoots, the content of oleanolic acid increased while the content of ursolic acid decreased (Fig. 14B). It can be concluded that chitosan elicitation partially enhanced the biosynthesis of some specialized metabolites (e.g., free pentacyclic acids) in *C. officinalis* plants; however, it negatively affected the biosynthesis of oleanolic acid saponins in shoots throughout the experiment, and in roots after 7 days (Fig. 15).

It was also determined that a single foliar chitosan application can produce effects long after treatment in plant organs that had not already developed, i.e., inflorescences. These results suggest that chitosan treatment substantially increased the biosynthesis of sterols in inflorescences (72%), and, less visibly and more variably, affected the content of triterpenoids (slightly increased free triterpenoids and decreased saponin content).

The results obtained in this study indicate that, in the case of at least some plants, a single treatment with chitosan might not result in desired enhancement of plant growth and fitness, though it may result in a metabolic rearrangement reflecting the trade-off between the general and specialized metabolites. However, the lack of effects on one plant species, particularly in comparison to many noted effects on other plant species, does not weaken its significance as an important biostimulant. Numerous reports of its positive role in alleviating abiotic stress have been published recently [43–46]. The present report is an important reminder that such results might not be universal and that an initial study on the chosen plant is recommended for the suitable selection of treatment conditions, including the dose and number of chitosan applications, the type of treatment (e.g., foliar or soil), and the vegetative stage of the treated plants.

## Conclusions

Chitosan is considered one of the most efficient biostimulants that can be applied to improve plant growth and yield, the content of bioactive specialized metabolites, and resistance to stress conditions and pathogens. It is widely accepted in contemporary agriculture as a non-toxic, biodegradable, and environment-friendly agent. The results obtained in this study confirmed the ability of chitosan to influence the growth and metabolism of the treated plant; however, the observed effects were not explicitly positive. Single foliar application of chitosan caused biomass reduction and did not increase the content of bioactive triterpenoid saponins in *Calendula officinalis* plants; moreover, similar effects were observed for chitosan-elicited hairy root cultures. Further studies are required to determine the effects of other variants of chitosan application exerted on the *C. officinalis* plant. Initial studies of the conditions of chitosan treatment are recommended before its application in agrotechnics to avoid unexpected underwhelming effects on crop plants.

## Materials and methods

### Hairy root cultures

*C. officinalis* hairy roots line CC16 (derived from cotyledon explant) was obtained according to a previously described procedure [47]. The roots were cultivated in a ½ Murashige–Skoog liquid medium, at 23–25 °C, in the darkness on a rotary shaker at 120 rpm. Subcultures were performed every 3–4 weeks by transferring the 1–2 cm pieces of the young branched root to 100 mL of a fresh medium.

### Pot cultures

*C. officinalis* seeds (PNOS, Ożarów Mazowiecki, Poland) were sown into rectangular plastic pots (dimensions: 80 × 18 × 14 cm) filled with universal flower soil “Athena” (the details of physical and chemical charaterization in Table S37). Germination of seed started within 3 days of sowing. The plants were cultivated for 3 weeks in the greenhouse under controlled conditions (16/8 h day/night fotoperiod, 52 ± 2% humidity, temperature 20 °C with a light intensity of 120 ± 10 µmol/m2*s).

### Chitosan elicitation of hairy roots

Freshly subcultured roots were incubated for 21 days to obtain at least 1.5 g of fresh weight. Afterwards, they were weighed and transferred to 100 ml of fresh medium five days prior to elicitation. Chitosan from crab shells (Cht, Sigma C3646, minimum 85% deacetylated) was prepared as described by Popp et al. (1997) [48]. The weighted sample was dissolved in 5% (v/v) HCl by gentle heating and continuous stirring. The pH of the solution was adjusted to 5.0 with 1 M NaOH. The final solution was autoclaved at 121 °C for 17 min and added to the culture medium to obtain a final concentration 50 mg/L. Control samples were supplemented with adequate volume of distilled water adjusted to pH 5.0 with 5% HCl. The cultures were cultured for 3, 7 and 14 days.

### Chitosan elicitation of pot-cultures

Three-weeks old marigold seedlings were sprayed with 50 mg/L chitosan solution until run-off (ca. 15 ml per plant). Control plants were sprayed with distilled water. The plants were cultivated either for 1 week or for 2 weeks in the greenhouse under conditions described above.

### Chlorophyll content determination

After 7 and 14 days of growing after chitosan treatment, chlorophyll was extracted from 20 mg of fresh plant tissue in 96% ethanol and determined by the method of Nair and Chung (2015) [49]. The absorbance of the supernatant was measured at wavelengths of 664.2 nm and 648.6 nm using a spectrophotometer. The total chlorophyll content (a and b) was determined by method developed by Lichtenthaler (1987) [50].

### Carotenoid content determination

Flowers from remaining plants were picked up during the blooming period starting approx. 1 month after elicitation. Freshly collected inflorescens were cut in half. One half was used to determine total carotenoid content, while the other half was left to dry in dark and airy space and used for further extractions described below. The total carotenoid were extracted from inflorescens with acetone, then the absorbance was measured at wavelength of 449 nm using Shimadzu UV-2401 PC spectrophotometer, and the total content was determined as described in [50].

### Extraction of the hairy roots and the culture medium

After 3, 7 and 14 days of incubation with chitosan, the culture media were filtered from the hairy roots. The harvested hairy roots were air-dried at room temperature before extraction, whereas the culture media were directly extracted 3 times with 40 mL portions of n-butanol to extract oleanolic acid saponins released to the medium. Dried hairy roots were powdered and extracted using a Soxhlet apparatus for 8 h with diethyl ether to obtain fractions of free sterols, conjugated sterols (esters and low polar glycosides), free triterpenoid acids and alcohols, and then 8 h with methanol to extract more polar sterol glycosides and oleanolic acid saponins. The obtained extracts were evaporated to dryness under reduced pressure on a rotary evaporator.

### Extraction of the plants

After 7 and 14 days of growing after chitosan treatment, plants were gently collected, then weighted, and lengths of the root and the shoots (aerial) parts were measured. Roots and aerial parts were left to dry in the dark and airy place, then weighted again. Dry roots, aerial parts and half of the inflorescences were then grinded in mortar to fine powder and extracted in Soxhlet apparatus for 8 h with diethyl ether and then 8 h with methanol. The obtained extracts were evaporated to dryness under reduced pressure on a rotary evaporator.

### Fractionation of diethyl ether extracts

Evaporated diethyl ether extracts obtained from the hairy roots, as well as roots and aerial parts of pot plants were fractionated by adsorption preparative TLC on 20 cm × 20 cm glass plates coated manually with silica gel 60H (Merck, Darmstadt, Germany). The solvent system chloroform: methanol 97:3 (v/v) was applied for developing. Four fractions were obtained as described by Sykłowska-Baranek et al. (2022): (i) esters; (ii) free steroids and neutral triterpenoids; (iii) free triterpenoid acids; and (iv) glycosides [51]. Fraction (ii) containing free steroids and neutral triterpenoids (alcohols) was directly analyzed using GC–MS, gas chromatography-mass spectrometer (Agilent Technologies 7890A); the triterpenoid acid fraction (iii) was methylated with diazomethane prior to GC–MS analysis as described previously [52]; the ester fraction (i) was subjected to alkaline hydrolysis to release the steroid core from ester forms; and the glycoside fraction (iv) to acidic hydrolysis to release the aglycones.

### Alkaline hydrolysis

The ester fractions were subjected to alkaline hydrolysis as decribed previously [22, 23] with 10% NaOH in 80% methanol at 80 °C for 3 h. Subsequently, 5 volumes of water were added to each hydrolysate, the pH was neutralized with 5% acetic acid, and the obtained mixtures were extracted with diethyl ether (3 × 20 mL) in a separation funnel. The extracts were evaporated and fractionated by preparative TLC to obtain the fraction of sterols released from their ester forms.

### Acidic hydrolysis

The glycoside fractions obtained from the diethyl ether extracts, evaporated methanol extracts and n-butanol extracts from the culture medium were hydrolyzed by 11% HCl in 80% methanol during 2 h on a heating mantle under reflux [51, 52]. Subsequently, the hydrolysates were diluted with distilled water, methanol was evaporated in a rotary evaporator, and the obtained aqueous remnants were extracted 3 times with 40 mL portions of diethyl ether in a separation funnel. The obtained extracts were washed with distilled water 3 times and evaporated to dryness.

### Fractionation of acidic hydrolysates

The dried extracts obtained from acidic hydrolysates were divided by preparative TLC on 20 cm × 20 cm glass plates, manually coated with silica gel 60H (Merck, Darmstadt, Germany). The solvent system chloroform: methanol 95:5 (v/v) was used for developing the plates. From hydrolysed methanol extracts two fractions were obtained: sterols and oleanolic acid. From hydrolysed n-butanol extracts only one fraction was obtained: oleanolic acid. Purified oleanolic acid was methylated with diazomethane.

### Derivatization of triterpenoid acids

Nitrosomethylurea (2.06 g) was added to a mixture of 20 mL of diethyl ether and 6 mL of 50% aqueous KOH, and the organic layer was then separated from the aqueous layer. Samples containing triterpenoid acids were dissolved in 2 mL of the obtained solution of diazomethane in diethyl ether, and held at 2° C for 24 h.

### Identification and quantification of triterpenoids by GC–MS/FID

An Agilent Technologies 7890 A gas chromatograph equipped with a 5975C mass spectrometric detector was used for qualitative and quantitative analyses. Samples dis-solved in diethyl ether:methanol (5:1, v/v) were applied (in a volume of 1–4 μl) using 1:10 split injection. The column used was a 30 m × 0.25 mm i.d., 0.25-μm, HP-5MS UI (Agilent Technologies, Santa Clara, CA). Helium was used as the carrier gas at a flow rate of 1 ml/min. The separation was made either under isothermal conditions at 280 °C or in the temperature programmed: initial temperature of 160 °C held for 2 min, then increased to 280 °C at 5 °C/1 min and the final temperature of 280 °C held for further 44 min. The other employed parameters were as follows: inlet and FID (flame ionization detector) temper-ature 290 °C; MS transfer line temperature 275 °C; quadrupole temperature 150 °C; ion source temperature 230 °C; EI 70 eV; m/z range 33–500; FID gas (H2) flow 30 ml•min − 1 (hydrogen generator); and air flow 400 ml min − 1. Individual compounds were identified by comparing their mass spectra with library data from Wiley 9th ED. and NIST 2008 Lib. SW Version 2010 or previously reported data and by comparison of their retention times and corresponding mass spectra with those of authentic standards, when available. Quantitation was performed using an external standard method based on calibration curves determined for the compounds belonging to representative triterpenoid classes: α-amyrin for triterpene alcohols, oleanolic acid methyl ester for triterpene acid methyl esters, and sitosterol for steroids.

### Statistical analysis of data

All data are presented as the means ± standard deviation of three independent samples analyzed in triplicate. To identify signifcant diferences between control and elicitor-treated samples, two-way analysis of variance (ANOVA) and the Tukey’s multiple comparisons test were applied (p ≤ 0.05). For the analysis of results obtained from inflorescences samples, T-student test was applied (p ≤ 0.05), as there was only one variable: treatment. Detailed statistical data are presented in supplementary materials. Statistical modeling was performed using the Statistica™ software (Version 13.3, © 1984–2017 TIBCO Software Inc).

## Supplementary Information

**Additional file 1: Table S1. **GC-MS data (retention times and characteristic ions of mass spectra) of identified steroids and triterpenoids. **Table S2.** Effect of chitosan treatment on steroids content in hairy roots tissue. Data which do not share a common letter are significantly different. Capital letters indicate significant difference in time between plants from the same treatment, lowercase indicate difference between treatments within certain time point. **Table S3.** Analysis of the interaction of treatment and time on steroid content in hairy roots tissue performed by two-way ANOVA. **Table S4.** Effect of chitosan treatment on sterol esters content in hairy roots tissue. Data which do not share a common letter are significantly different. Capital letters indicate significant difference in time between plants from the same treatment, lowercase indicate difference between treatments within certain time point. **Table S5.** Effect of chitosan treatment on sterol glycosides content in hairy roots tissue. Data which do not share a common letter are significantly different. Capital letters indicate significant difference in time between plants from the same treatment, lowercase indicate difference between treatments within certain time point. **Table S6.** Analysis of the interaction of treatment and time on sterol esters and sterol glycosides content in hairy roots tissue performed by two-way ANOVA. **Table S7.** Effect of chitosan treatment on neutral terpenoids content in hairy roots tissue. Data which do not share a common letter are significantly different. Capital letters indicate significant difference in time between plants from the same treatment, lowercase indicate difference between treatments within certain time point. **Table S8.** Analysis of the interaction of treatment and time on neutral triterpenoids (amyrins) content in hairy roots tissue performed by two-way ANOVA. **Table S9.** Effect of chitosan treatment on free oleanolic acid (OA) and its methyl ester (Met OA) content in hairy roots tissue. Data which do not share a common letter are significantly different. Capital letters indicate significant difference in time between plants from the same treatment, lowercase indicate difference between treatments within certain time point. **Table S10.** Analysis of the interaction of treatment and time on free oleanolic acid (OA) and its methyl ester (Met OA) content in hairy roots tissue performed by two-way ANOVA. **Table S11.** Effect of chitosan treatment on oleanolic acid saponins (OA) content in hairy roots tissue. Data which do not share a common letter are significantly different. Capital letters indicate significant difference in time between plants from the same treatment, lowercase indicate difference between treatments within certain time point. **Table S12.** Analysis of the interaction of treatment and time on oleanolic acid (OA) saponins content in hairy roots tissue performed by two-way ANOVA. **Table S13.** Effect of chitosan treatment on oleanolic acid saponins (OA) released to the culture medium. Data which do not share a common letter are significantly different. Capital letters indicate significant difference in time between plants from the same treatment, lowercase indicate difference between treatments within certain time point. **Table S14.** Analysis of the interaction of treatment and time on oleanolic acid (OA) saponins released to the medium performed by two-way ANOVA. **Table S15.** Content of free sterols, neutral triterpenoids and triterpenoid acids in C. officinalis roots. Data which do not share a common letter are significantly different. Capital letters indicate significant difference in time between plants from the same treatment, lowercase indicate difference between treatments within certain time point. **Table S16.** Analysis of the interaction of treatment and time on free sterols content in C. officinalis roots performed by two-way ANOVA. **Table S17.** Analysis of the interaction of treatment and time on neutral triterpenoids (amyrins and friedooleanans) content in C. officinalis roots performed by two-way ANOVA. **Table S18.** Analysis of the interaction of treatment and time on triterpenoid acids content in C. officinalis roots performed by two-way ANOVA. **Table S19.** Content of sterols conjugated in sterol esters and sterol glycosides in C. officinalis roots. Data which do not share a common letter are significantly different. Capital letters indicate significant difference in time between plants from the same treatment, lowercase indicate difference between treatments within certain time point. **Table S20.** Analysis of the interaction of treatment and time on sterol esters and sterol glycosides content in C. officinalis roots performed by two-way ANOVA. **Table S21.** Content of free sterols, neutral triterpenoids and triterpenoid acids in C. officinalis shoots. Data which do not share a common letter are significantly different. Capital letters indicate significant difference in time between plants from the same treatment, lowercase indicate difference between treatments within certain time point. **Table S22.** Analysis of the interaction of treatment and time on free sterols content in C. officinalis shoots performed by two-way ANOVA. **Table S23.** Analysis of the interaction of treatment and time on neutral triterpenoids (amyrins) content in C. officinalis shoots performed by two-way ANOVA. **Table S24.** Analysis of the interaction of treatment and time on triterpenoid acids content in C. officinalis shoots performed by two-way ANOVA. **Table S25.** Content of sterols conjugated in sterol esters and sterol glycosides in C. officinalis shoots. Data which do not share a common letter are significantly different. Capital letters indicate significant difference in time between plants from the same treatment, lowercase indicate difference between treatments within certain time point. **Table S26.** Analysis of the interaction of treatment and time on sterol esters and sterol glycosides content in C. officinalis shoots performed by two-way ANOVA. **Table S27.** Content of oleanolic acid saponins (OA) in C. officinalis roots and shoots. Data which do not share a common letter are significantly different. Capital letters indicate significant difference in time between plants from the same treatment, lowercase indicate difference between treatments within certain time point. **Table S28.** Analysis of the interaction of treatment and time on oleanolic acid (OA) saponins content in roots and shoots of C. officinalis performed by two-way ANOVA. **Table S29.** Content of free sterols, neutral triterpenoids and triterpenoid acids in C. officinalis inflorescences. Data which do not share a common letter are significantly different. **Table S30.** Analysis of the chitosan treatment on free sterols content in C. officinalis inflorescences performed by T- student test. **Table S31.** Analysis of the chitosan treatment on neutral triterpenoids content in C. officinalis inflorescences performed by T- student test. **Table S32.** Analysis of the chitosan treatment on free oleanolic acid (OA) content in C. officinalis inflorescences performed by T- student test. **Table S33.** Content of sterols conjugated in sterol glycosides in C. officinalis inflorescences. Data which do not share a common letter are significantly different. **Table S34.** Analysis of the chitosan treatment on sterol glycosides content in C. officinalis inflorescences performed by T- student test. **Table S35.** Content of oleanolic acid (OA) saponins in C. officinalis inflorescences. Data which do not share a common letter are significantly different. **Table S36.** Analysis of the chitosan treatment on oleanolic acid (OA) saponins content in C. officinalis inflorescences performed by T- student test. **Table S37.** Basic physical and chemical charactrerization of universal soil “Athena” including: pH, salinity, concentration of nitrogen (N), potassium oxide (K2O) and phosphates (P2O5).

## Data Availability

The datasets used and/or analysed during the current study are available from the corresponding author on reasonable request.
