# Supplementary material for: Modifications in steroid and triterpenoid metabolism in Calendula officinalis plants and hairy root culture in response to chitosan treatment
Source: BMC Plant Biol. 2023 May 18;23:263. doi: 10.1186/s12870-023-04261-4 (PMC10193699; doi:10.1186/s12870-023-04261-4)
Supplement: Supplementary file 1 — Additional file 1: Table S1. GC-MS data (retention times and characteristic ions of mass spectra) of identified steroids and triterpenoids. Table S2. Effect of chitosan treatment on steroids content in hairy roots tissue. Data which do not share a common letter are significantly different. Capital letters indicate significant difference in time between plants from the same treatment, lowercase indicate difference between treatments within certain time point. Table S3. Analysis of the interaction of treatment and time on steroid content in hairy roots tissue performed by two-way ANOVA. Table S4. Effect of chitosan treatment on sterol esters content in hairy roots tissue. Data which do not share a common letter are significantly different. Capital letters indicate significant difference in time between plants from the same treatment, lowercase indicate difference between treatments within certain time point. Table S5. Effect of chitosan treatment on sterol glycosides content in hairy roots tissue. Data which do not share a common letter are significantly different. Capital letters indicate significant difference in time between plants from the same treatment, lowercase indicate difference between treatments within certain time point. Table S6. Analysis of the interaction of treatment and time on sterol esters and sterol glycosides content in hairy roots tissue performed by two-way ANOVA. Table S7. Effect of chitosan treatment on neutral terpenoids content in hairy roots tissue. Data which do not share a common letter are significantly different. Capital letters indicate significant difference in time between plants from the same treatment, lowercase indicate difference between treatments within certain time point. Table S8. Analysis of the interaction of treatment and time on neutral triterpenoids (amyrins) content in hairy roots tissue performed by two-way ANOVA. Table S9. Effect of chitosan treatment on free oleanolic acid (OA) and its methyl ester (Met OA) content [file 12870_2023_4261_MOESM1_ESM.pdf]

Modifications in steroid and triterpenoid metabolism in Calendula officinalis plants and hairy root culture in response to chitosan treatment

Table S1.GC-MS data (retention times and characteristic ions of mass spectra) of identified steroids and triterpenoids.

Compound	Formula	Molecular weight	Retention time[min]	Mass spectrum *m/z*(relative intensity)
cholesterol	$C_{27}H_{46}O$	386.6	31.06	386 (26), 107 (50), 105 (48), 91 (57), 81 (54), 79 (46), 69 (47), 57 (87), 55 (73), 43 (100), 41 (55)
campesterol	$C_{28}H_{48}O$	400.6	33.59	400 (30), 107 (51), 105 (55), 95 (49), 83 (45), 81 (64), 71 (62), 57 (77), 55 (77), 43 (100), 41 (52)
stigmasterol	$C_{29}H_{48}O$	412.6	34.52	412 (36), 145 (64), 107 (52), 95 (100), 83 (66), 81 (90), 78 (60), 69 (67), 67 (85), 55 (69)
sitosterol	$C_{29}H_{50}O$	414.7	36.15	414 (29), 145 (54), 107 (59), 105 (60), 95 (54), 91 (49), 81 (57), 57 (68), 55 (70), 43 (100)
sitostanol	$C_{29}H_{52}O$	416.7	36.40	416 (31), 215 (82), 109 (58), 107 (83), 95 (81), 93 (64), 81 (84), 69 (60), 57 (64), 55 (81), 43 (100)
isofucosterol	$C_{29}H_{48}O$	412.3	36.78	412 (5), 314 (100), 105 (47), 95 (50), 91 (42), 83 (40), 81 (51), 69 (61), 55 (96), 43 (49)
cycloartanol	$C_{30}H_{52}O$	428.7	36.90	428 (4), 205 (60), 109 (98), 95 (100), 93 (64), 81 (69), 69 (78), 57 (73), 55 (82), 43 (89), 41 (67)
β-amyrin	$C_{30}H_{50}O$	426.7	37.13	426 (27), 219 (18), 218 (100), 203 (49), 189 (17), 135 (11), 109 (13), 105 (12), 95 (15), 81 (18), 69 (14)
α-amyrin	$C_{30}H_{50}O$	426.7	38.62	426 (4), 219 (18), 218 (100), 203 (20), 189 (19), 135 (17), 133 (15), 122 (16), 119 (15), 95 (16)
tremulone	$C_{29}H_{46}O$	410.7	39.20	410 (32), 187 (27), 174 (100), 161 (37), 159 (26), 91 (28), 57 (28), 55 (37), 43 (44), 41 (28)
24-methylene-cycloartanol	$C_{31}H_{52}O$	440.7	40.50	440 (5), 121 (60), 119 (55), 109 (62), 107 (76), 105 (57), 95 (98), 93 (64), 81 (72), 69 (99), 55 (100)
sitostenone	$C_{29}H_{48}O$	412.7	40.72	412 (37), 229 (34), 218 (31), 124 (100), 109 (31), 95 (41), 81 (27), 69 (32), 55 (37), 43 (44)
cycloartenol acetate	$C_{32}H_{52}O_2$	469.8	41.3	468 (24), 121 (20), 109 (32), 107 (29), 95 (41), 93 (24), 81 (27), 69 (30), 55 (23), 43 (100)
ψ-taraxasterol	$C_{30}H_{50}O$	426.7	41.82	426 (21), 207 (65), 189 (100), 135 (50), 121 (54), 119 (29), 109 (39), 107 (40), 95 (70), 93 (48)
taraxasterol	$C_{30}H_{50}O$	426.7	42.25	426 (33), 207 (100), 205 (34), 191 (39), 190 (45), 189 (87), 135 (48), 121 (30), 109 (31), 107 (37)
friedelinol	$C_{30}H_{52}O$	428.7	42.7	428 (3), 125 (45), 123 (49), 121 (48), 109 (75), 107 (47), 96 (68), 95 (100), 81 (66), 69 (82)
friedelin	$C_{30}H_{50}O$	426.7	43.7	426 (6), 125 (65), 123 (78), 109 (82), 107 (46), 96 (62), 95 (94), 81 (77), 69 (100), 67 (56)
oleanolic acid methyl ester	$C_{31}H_{50}O_3$	470.1	46.37	470 (1), 262 (48), 207 (13), 204 (16), 203 (100), 202 (21), 189 (22), 133 (17), 119 (13), 105 (14

stigmastane-3,6-dione	$C_{29}H_{48}O_2$	428.6	48.03	428 (25), 135 (61), 107 (74), 98 (63), 95 (67), 79 (62), 69 (86), 57 (67), 55 (100), 43 (77), 41 (71)
ursolic acid methyl ester	$C_{31}H_{50}O_3$	470.1	48.96	470 (1), 263 (20), 262 (100), 207 (32), 203 (93), 189 (29), 133 (76), 119 (34), 105 (21), 95 (18)
faradiol	$C_{30}H_{50}O_2$	442.7	58.50	442 (25), 207 (78), 189 (100), 135 (65), 123 (60), 121 (80), 119 (59), 109 (74), 107 (77), 95 (91)

Table S2. Effect of chitosan treatment on steroids content in hairy roots tissue. Data which do not share a common letter are significantly different. Capital letters indicate significant difference in time between plants from the same treatment, lowercase indicate difference between treatments within certain time point.

Compound	Content [µg/g DW± SD]					
	days					
	3		7		14	
	C	CH	C	CH	C	CH
cholesterol	5.18±0.85 A, a	15.40±2.09 A, b	5.28±0.64 A, a	7.13±0.99 B, b	5.63±0.98 A, a	5.49±0.76 B, b
campesterol	27.33±2.75A, a	21.75±2.03 A, a	53.44±3.98 B, a	19.84±0.48 A, b	80.01±8.48 C, a	19.06±1.58 A, b
stigmasterol	168.76±14.81 A,a	41.69±3.08 A, b	517.89±38.39 B, a	41.94±5.63 A, b	958.88±44.58 C, a	27.26±2.82 A, b
sitosterol	144.93±6.51 A, a	137.15±12.41 A, a	168.24±14.81 A, a	138.05±12.42 A, a	154.58±19.56 A, a	120.55±18.74 A, a
sitostanol	17.58±1.77 A, a	37.72±6.63 A, b	20.23±3.51 A, a	48.91±4.56 A, b	18.77±2.29 A, a	46.33±5.38 A, b
izofucosterol	22.22±2.44 A, a	n.d. A, b	10.05±1.69 B, a	n.d. A, b	30.31±2.97 C, a	n.d. A, b
cycloartanol	n.d. A, a	27.51±1.75 A, b	n.d. A, a	29.37±4.05 A, b	n.d. A, a	23.88±4.95 A, b
tremulone	33.20±3.15 A, a	42.33±5.76 A, a	14.97±1.89 B, a	47.81±6.02 A, b	5.94±0.98 B, a	43.81±3.01 A, b
24-methylene cycloartenol	18.94±1.13 A, a	16.51±1.44 A, a	16.59±2.79 A, a	25.95±1.33 B, b	14.52±2.45 A, a	8.41±0.63 C, b
**Total**	**438.14**	**340.06**	**806.70**	**359.01**	**1268.64**	**294.80**

Table S3. Analysis of the interaction of treatment and time on steroid content in hairy roots tissue performed by two-way ANOVA.

	***p* value**							
	cholesterol	campesterol	stigmasterol	sitosterol	sitostanol	isofucosterol	tremulone	24-methylenecycloartanol
treatment	<0.001	<0.001	<0.001	0.005	<0.001	<0.001	<0.001	n.s.
time	<0.001	<0.001	<0.001	n.s.	0.047	n.s.	<0.001	<0.001
treatment x time	<0.001	<0.001	<0.001	n.s.	n.s.	n.s.	<0.001	<0.001

Table S4. Effect of chitosan treatment on sterol esters content in hairy roots tissue. Data which do not share a common letter are significantly different. Capital letters indicate significant difference in time between plants from the same treatment, lowercase indicate difference between treatments within certain time point.

Compound	Content [μg/g DW± SD]					
	days					
	3		7		14	
	C	CH	C	CH	C	CH
cholesterol	11.84±1.56 A, a	46.97±4.30 A, b	18.17±1.71 A, a	50.35±6.68 A, b	8.74±1.57 A, a	47.11±3.24 A, b
campesterol	14.81±1.06 AB, a	36.69±2.66 A, b	21.83±1.21 A, a	66.45±7.22 B, b	8.38±0.99 B, a	61.61±4.02 B, b
stigmasterol	28.39±2.98 A, a	49.36±4.29 A, b	27.98±2.14 A, a	40.71±2.49 A, b	30.68±4.00 A, a	41.24±3.60 A, b
sitosterol	40.33±3.77 A, a	84.31±6.54 A, b	58.55±5.01 B, a	89.90±6.52 A, b	29.06±3.74 A, a	129.65±8.55 B, b
**Total**	**95.37**	**217.34**	**126.53**	**247.40**	**76.86**	**279.61**

Table S5. Effect of chitosan treatment on sterol glycosides content in hairy roots tissue. Data which do not share a common letter are significantly different. Capital letters indicate significant difference in time between plants from the same treatment, lowercase indicate difference between treatments within certain time point.

Compound	Content [μg/g DW± SD]					
	days					
	3		7		14	
	C	CH	C	CH	C	CH
cholesterol	11.24±1.19 A, a	11.33±2.51 A, a	10.37±1.04 AB, a	10.57±0.96 A, a	6.73±0.56 B, a	21.70±2.45 B, b
campesterol	66.00±5.46 A, a	15.77±1.42 A, b	21.41±2.31 B, a	13.09±2.86 A, a	18.12±1.23 B, a	36.38±3.72 B, b
stigmasterol	117.90±7.87 A, a	59.01±4.71 A, b	76.52±5.80 B, a	80.16±6.05 B, a	75.38±6.71 B, a	44.73±8.66 A, b
sitosterol	83.37±7.65 A, a	98.15±5.17 A, b	78.30±4.91 A, a	73.31±5.68 B, a	39.50±3.10 B, a	53.95±4.60 C, a
**Total**	**278.51**	**184.26**	**186.60**	**177.13**	**139.73**	**156.77**

Table S6. Analysis of the interaction of treatment and time on sterol esters and sterol glycosides content in hairy roots tissue performed by two-way ANOVA.

	***p* value**			
	cholesterol	campesterol	stigmasterol	sitosterol
**Sterol esters**				
treatment	<0.001	<0.001	<0.001	<0.001
time	0.029	<0.001	n.s.	0.001
treatment x time	n.s.	<0.001	0.045	<0.001
**Sterol glycosides**				
treatment	<0.001	<0.001	<0.001	0.008
time	0.005	<0.001	<0.001	<0.001
treatment x time	<0.001	<0.001	<0.001	0.011

Table S7. Effect of chitosan treatment on neutral terpenoids content in hairy roots tissue. Data which do not share a common letter are significantly different. Capital letters indicate significant difference in time between plants from the same treatment, lowercase indicate difference between treatments within certain time point.

**Compound**	**Content [µg/g DW± SD]**					
	**days**					
	3		7		14	
	C	CH	C	CH	C	CH
β-amyrin	11.40±0.77 A, a	14.30±1.59 A, a	8.79±0.71 A, a	37.13±4.85 B, b	5.65±0.97 A, a	15.72±2.56 A, b
α-amyrin	15.95±1.14 A, a	30.55±1.83 A, b	16.89±3.06 A, a	34.65±2.55 A, b	18.87±3.23 A, a	18.79±2.35 B, a
**Total:**	**27.34**	**44.85**	**25.68**	**71.78**	**24.52**	**34.51**

Table S8. Analysis of the interaction of treatment and time on neutral triterpenoids **(**amyrins) content in hairy roots tissue performed by two-way ANOVA.

	***p* value**	
	β-amyrin	α-amyrin
treatment	<0.001	<0.001
time	<0.001	0.001
treatment x time	<0.001	<0.001

Table S9. Effect of chitosan treatment on free oleanolic acid (OA) and its methyl ester (Met OA) content in hairy roots tissue. Data which do not share a common letter are significantly different. Capital letters indicate significant difference in time between plants from the same treatment, lowercase indicate difference between treatments within certain time point.

**Compound**	**Content [µg/g DW± SD]**					
	**days**					
	3		7		14	
	C	CH	C	CH	C	CH
OA	13.22±0.31 A, a	17.80±1.23 A, b	13.83±1.64 A, a	18.24±1.39 A, b	13.51±1.65 A, a	19.33±1.21 A, b
Met OA	n.d. A, a	65.40±4.78 A, b	n.d. A, a	48.88±4.33 B, b	n.d. A, a	35.31±5.40 C, b

Table S10. Analysis of the interaction of treatment and time on free oleanolic acid (OA) and its methyl ester (Met OA) content in hairy roots tissue performed by two-way ANOVA.

	***p* value**	
	OA	Met OA
treatment	<0.001	<0.001
time	n.s.	<0.001
treatment x time	n.s.	<0.001

Table S11. Effect of chitosan treatment on oleanolic acid saponins (OA) content in hairy roots tissue. Data which do not share a common letter are significantly different. Capital letters indicate significant difference in time between plants from the same treatment, lowercase indicate difference between treatments within certain time point.

**Compound**	**Content [µg/g DW± SD]**					
	**days**					
	3		7		14	
	C	CH	C	CH	C	CH
OA	1870.22±100.47 A, a	1343.19±218.31 A, a	3317.20±260.72 B, a	795.07±96.78 AB, a	4830.75±574.58 C, a	45.46±4.39 B, b

Table S12. Analysis of the interaction of treatment and time on oleanolic acid (OA) saponins content in hairy roots tissue performed by two-way ANOVA.

	***p* value**
	OA
treatment	<0.001
time	0.001
treatment x time	<0.001

Table S13. Effect of chitosan treatment on oleanolic acid saponins (OA) released to the culture medium. Data which do not share a common letter are significantly different. Capital letters indicate significant difference in time between plants from the same treatment, lowercase indicate difference between treatments within certain time point.

**Compound**	**Content [mg/L•g DW± SD]**					
	**days**					
	3		7		14	
	C	CH	C	CH	C	CH
OA	0.58±0.04 A, a	0.58±0.09 A, a	0.59±0.05 A, a	0.86±0.04 B, b	0.80±0.16 A, a	0.54±0.10 A, b

Table S14. Analysis of the interaction of treatment and time on oleanolic acid (OA) saponins released to the medium performed by two-way ANOVA.

	***p* value**
	OA
treatment	n.s.
time	n.s.
treatment x time	0.001

Table S15. Content of free sterols, neutral triterpenoids and triterpenoid acids in *C. officinalis* roots. Data which do not share a common letter are significantly different. Capital letters indicate significant difference in time between plants from the same treatment, lowercase indicate difference between treatments within certain time point.

**Compound**	**Content [μg/g DW± SD]**			
	**Days**			
	7		14	
	C	CH	C	CH
**Free sterols:**				
cholesterol	11.32±0.55 A, a	5.65±0.41 A, b	6.41±0.83 B, a	8.28±0.61 B, b
campesterol	84.75±6.91 A, a	67.96±1.31 A, b	50.89±2.41 B, a	29.39±1.34 B, b
stigmasterol	804.98±27.26 A, a	415.88±18.40 A, b	433.79±50.21 B, a	238.60±25.34 B, b
sitosterol	446.97±41.21 A, a	472.90±18.72 A, a	316.98±24.32 B, a	131.68±10.54 B, b
sitostanol	122.45±12.46 A, a	130.21±8.15 A, a	62.63±7.26 B, a	73.50±1.46 B, a
tremulone	32.16±2.72 A, a	35.97±2.06 A, a	10.50±0.82 B, a	20.94±0.75 B, b
sitostenone	115.82±9.30 A, a	116.46±5.10 A, a	48.34±12.21 B, a	34.15±1.02 B, a
cycloartenol acetate	21.46±1.65 A, a	24.88±0.80 A, b	14.79±1.21 B, a	11.19±0.89 B, b
stigmastan-3,6-dione	n.d. A, a	15.45±1.25 A, b	11.15±2.75 B, a	18.97±0.45 A, b
**Total sterols:**	**1639.90**	**1285.35**	**955.49**	**566.68**
**Neutral triterpenoids:**				
β-amyrin	47.52±4.89 A, a	46.07±1.09 A, a	31.70±1.93 B, a	28.16±0.61 B, a
α-amyrin	38.56±7.91 A, a	43.92±1.34 A, a	30.81±2.02 A, a	24.24±1.44 B, a
**Sum of amyrins:**	**86.08**	**89.98**	**62.52**	**52.40**
fridelinol	77.05±7.70 A, a	24.40±1.86 A, b	80.42±16.54 A, a	21.81±1.98 A, b
friedelin	44.76±4.88 A, a	11.23±1.41 A, b	49.37±3.34 A, a	13.14±2.67 A, b
**Sum of friedooleanans:**	**121.81**	**35.62**	**129.79**	**34.95**
**Triterpenoid acids:**				
OA	19.80±0.88 A, a	37.26±1.74 A, b	23.45±2.67 A, a	32.04±2.32 A, b
UA	32.92±3.98 A, a	58.48±5.31 A, b	44.84±4.81 B, a	43.22±2.98 B, a
**Sum of triterpenoid acids:**	**52.72**	**95.75**	**68.29**	**75.26**

Table S16. Analysis of the interaction of treatment and time on free sterols content in *C. officinalis* roots performed by two-way ANOVA.

	***p* value**								
	cholesterol	campesterol	stigmasterol	sitosterol	sitostanol	tremulone	sitostenone	cycloartenol acetate	stigmastan-3,6-dione
treatment	<0.001	<0.001	<0.001	0.001	n.s.	<0.001	n.s.	n.s.	<0.001
time	0.013	<0.001	<0.001	<0.001	<0.001	<0.001	<0.001	<0.001	<0.001
treatment x time	<0.001	n.s.	0.001	<0.001	n.s.	0.013	n.s.	0.001	0.003

Table S17. Analysis of the interaction of treatment and time on neutral triterpenoids (amyrins and friedooleanans) content in *C. officinalis* roots performed by two-way ANOVA.

	***p* value**			
	β-amyrin	α-amyrin	fridelinol	friedelin
treatment	n.s.	n.s.	<0.001	<0.001
time	<0.001	<0.001	n.s.	n.s.
treatment x time	n.s.	0.039	n.s.	n.s.

Table S18. Analysis of the interaction of treatment and time on triterpenoid acids content in *C. officinalis* roots performed by two-way ANOVA.

	***p* value**	
	OA	UA
treatment	<0.001	0.001
time	n.s.	n.s.
treatment x time	0.005	0.001

Table S19. Content of sterols conjugated in sterol esters and sterol glycosides in *C. officinalis* roots. Data which do not share a common letter are significantly different. Capital letters indicate significant difference in time between plants from the same treatment, lowercase indicate difference between treatments within certain time point.

**Compound**	**Content [µg/g DW± SD]**			
	**Days**			
	7		14	
	C	CH	C	CH
**Sterol esters:**				
cholesterol	50.28±1.01 A, a	35.27±4.48 A, b	16.65±0.91 B, a	27.40±4.49 A, b
campesterol	29.45±2.28 A, a	53.01±6.14 A, b	23.35±1.51 A, a	32.88±3.13 B, a
stigmasterol	22.98±2.09 A, a	38.49±4.88 A, b	22.83±1.51 A, a	26.60±0.60 B, a
sitosterol	31.49±3.02 A, a	55.15±6.21 A, b	21.43±2.92 A, a	32.65±3.99 B, b
**Total:**	**134.21**	**181.93**	**84.27**	**119.52**
**Sterol glycosides:**				
cholesterol	42.87±9.54 A, a	26.03±2.05 A, b	5.59±0.37 B, a	10.64±1.20 B, a
campesterol	43.33±5.07 A, a	95.16±10.18 A, b	18.41±2.86 B, a	41.69±6.63 B, b
stigmasterol	47.29±2.84 A, a	45.07±2.56 A, a	24.52±2.70 B, a	17.75±1.34 B, b
sitosterol	104.69±6.39 A, a	98.41±3.37 A, a	46.73±5.23 B, a	70.83±3.41 B, b
**Total:**	**238.19**	**264.67**	**95.25**	**140.91**

Table S20. Analysis of the interaction of treatment and time on sterol esters and sterol glycosides content in *C. officinalis* roots performed by two-way ANOVA.

	***p* value**			
	cholesterol	campesterol	stigmasterol	sitosterol
**Sterol esters**				
treatment	n.s.	<0.001	<0.001	<0.001
time	<0.001	<0.001	0.006	<0.001
treatment x time	<0.001	0.011	0.006	0.035
**Sterol glycosides**				
treatment	n.s.	<0.001	0.013	0.012
time	<0.001	<0.001	<0.001	<0.001
treatment x time	0.005	0.006	n.s.	0.001

Table S21. Content of free sterols, neutral triterpenoids and triterpenoid acids in *C. officinalis* shoots. Data which do not share a common letter are significantly different. Capital letters indicate significant difference in time between plants from the same treatment, lowercase indicate difference between treatments within certain time point.

**Compound**	**Content [μg/g DW± SD]**			
	**Days**			
	7		14	
	C	CH	C	CH
**Free sterols:**				
cholesterol	9.15±1.48 A, a	9.62±0.38 A, a	16.83±3.54 B, a	4.49±1.13 A, b
campesterol	26.07±4.56 A, a	29.87±4.23 A, a	43.66±0.67 B, a	13.79±0.39 B, b
stigmasterol	385.75±82.44 A, a	474.68±45.51 A, a	390.37±32.24 A, a	253.78±14.09 B, b
sitosterol	215.58±29.77 A, a	244.01±19.82 A, a	154.06±9.90 B, a	121.67±2.38 B, a
sitostanol	16.87±2.69 A, a	14.54±2.64 A, a	19.85±6.79 A, a	12.21±2.73 A, a
tremulone	6.05±1.64 A, a	7.75±1.95 A, a	7.97±2.94 A, a	6.33±1.87 A, a
sitostenone	13.48±4.45 A, a	15.62±4.38 A, a	13.47±2.16 A, a	5.88±0.63 B, a
**Total sterols:**	**672.96**	**796.10**	**646.21**	**418.15**
**Neutral triterpenoids:**				
β-amyrin	39.83±2.81 A, a	50.00±1.16 A, b	38.11±4.15 A, a	33.59±3.79 B, a
α-amyrin	50.35±6.97 A, a	88.85±1.01 A, b	53.03±6.74 A, a	104.69±6.20 B, b
**Sum of amyrins:**	**90.18**	**138.85**	**91.14**	**138.29**
**Triterpenoid acids:**				
OA	8.11±1.46 A, a	22.33±1.19 A, b	16.34±1.04 B, a	21.13±0.23 A, b
UA	24.72±2.55 A, a	13.73±1.44 A, b	19.81±1.48 B, a	8.96±1.12 B, b
**Sum of triterpenoid acids:**	**32.82**	**36.06**	**36.15**	**30.10**

Table S22. Analysis of the interaction of treatment and time on free sterols content in *C. officinalis* shoots performed by two-way ANOVA.

	***p* value**						
	cholesterol	campesterol	stigmasterol	sitosterol	sitostanol	tremulone	sitostenone
treatment	0.001	<0.001	n.s.	n.s.	n.s.	n.s.	n.s.
time	n.s.	n.s.	0.006	<0.001	n.s.	n.s.	0.034
treatment x time	0.001	<0.001	0.005	0.022	n.s.	n.s.	0.035

Table S23. Analysis of the interaction of treatment and time on neutral triterpenoids (amyrins) content in *C. officinalis* shoots performed by two-way ANOVA.

	***p* value**	
	β-amyrin	α-amyrin
treatment	n.s.	<0.001
time	0.001	0.024
treatment x time	0.004	n.s.

Table S24. Analysis of the interaction of treatment and time on triterpenoid acids content in *C. officinalis* shoots performed by two-way ANOVA.

	***p* value**	
	OA	UA
treatment	<0.001	0.001
time	n.s.	n.s.
treatment x time	0.005	0.001

Table S25. Content of sterols conjugated in sterol esters and sterol glycosides in *C. officinalis* shoots. Data which do not share a common letter are significantly different. Capital letters indicate significant difference in time between plants from the same treatment, lowercase indicate difference between treatments within certain time point.

**Compound**	**Content [µg/g DW± SD]**			
	**Days**			
	7		14	
	C	CH	C	CH
**Sterol esters:**				
cholesterol	22.57±4.99 A, a	7.86±0.84 A, b	14.26±2.43 B, a	9.32±0.47 A, a
campesterol	29.35±1.90 A, a	31.20±4.09 A, a	11.65±2.29 B, a	16.60±0.88 B, a
stigmasterol	16.67±2.37 A, a	16.31±1.32 A, a	15.17±1.37 A, a	12.47±2.08 A, a
sitosterol	36.73±2.33 A, a	81.89±6.52 A, b	12.99±1.16 B, a	30.54±2.61 B, b
**Total:**	**105.31**	**137.26**	**54.07**	**68.94**
**Sterol glycosides:**				
cholesterol	7.65±2.29 A, a	21.47±1.82 A, b	7.77±0.46 A, a	11.35±2.10 B, a
campesterol	21.22±2.38 A, a	24.52±3.38 A, a	21.47±2.87 A, a	22.02±2.25 A, a
stigmasterol	79.70±6.78 A, a	38.33±1.36 A, b	143.77±2.95 B, a	157.22±7.74 B, a
sitosterol	73.29±8.55 A, a	41.74±2.83 A, b	107.17±6.18 B, a	131.86±13.09 B, b
**Total:**	**181.86**	**126.06**	**280.18**	**322.45**

Table S26. Analysis of the interaction of treatment and time on sterol esters and sterol glycosides content in *C. officinalis* shoots performed by two-way ANOVA.

	***p* value**			
	cholesterol	campesterol	stigmasterol	sitosterol
**Sterol esters**				
treatment	<0.001	n.s.	n.s.	<0.001
time	n.s.	<0.001	0.036	<0.001
treatment x time	0.017	n.s.	n.s.	<0.001
**Sterol glycosides**				
treatment	<0.001	n.s.	0.002	n.s.
time	0.001	n.s.	<0.001	<0.001
treatment x time	0.001	n.s.	<0.001	<0.001

Table S27. Content of oleanolic acid saponins (OA) in *C. officinalis* roots and shoots. Data which do not share a common letter are significantly different. Capital letters indicate significant difference in time between plants from the same treatment, lowercase indicate difference between treatments within certain time point.

**Organ**	**Content [µg/g DW± SD]**			
	**Days**			
	7		14	
	C	CH	C	CH
**Roots**				
OA	2474.64±205.76 A, a	698.76±63.50 A, b	4233.38±298.04 B, a	8926.08±206.57 B, b
**Shoots**				
OA	11588.57±855.98 A, a	8549.61±264.95 A, b	18818.27±360.98 B, a	14886.09±474.71 B, b

Table S28. Analysis of the interaction of treatment and time on oleanolic acid (OA) saponins content in roots and shoots of *C. officinalis* performed by two-way ANOVA.

	***p* value**	
	OA roots	OA shoots
treatment	<0.001	<0.001
time	<0.001	<0.001
treatment x time	<0.001	n.s.

Table S29. Content of free sterols, neutral triterpenoids and triterpenoid acids in *C. officinalis* inflorescences. Data which do not share a common letter are significantly different.

**Compound**	**Content [µg/g DW± SD]**	
	**C**	**CH**
**Free sterols:**		
campesterol	85.81±0.91 a	281.08±17.88 b
stigmasterol	388.86±18.52 a	590.24±16.13 b
sitosterol	247.02±29.21 a	400.10±9.23 b
isofucosterol	281.49±16.44 a	450.06±7.21 b
**Total sterols:**	**1003.17**	**1721.48**
**Neutral triterpenoids:**		
β-amyrin	2195.27±104.88 a	2712.86±248.27 a
α-amyrin	1576.42±76.29 a	1944.34±242.37 a
ψ-taraxasterol	3773.87±136.03 a	4050.13±479.73 a
taraxasterol	560.34±33.64 a	707.43±98.50 a
faradiol	34.64±4.97 a	35.80±3.74 a
**Sum of neutral triterpenoids:**	**8140.54**	**9450.55**
**Triterpenoid acids:**		
OA	154.80±15.01 a	148.66±12.44 a

Table S30. Analysis of the chitosan treatment on free sterols content in *C. officinalis* inflorescences performed by T- student test.

	***p* value**			
	campesterol	stigmasterol	sitosterol	isofucosterol
treatment	<0.001	<0.001	0.007	<0.001

Table S31. Analysis of the chitosan treatment on neutral triterpenoids content in *C. officinalis* inflorescences performed by T- student test.

	***p* value**				
	β-amyrin	α-amyrin	ψ-taraxasterol	taraxasterol	faradiol
treatment	n.s.	n.s.	n.s.	n.s.	n.s.

Table S32. Analysis of the chitosan treatment on free oleanolic acid (OA) content in *C. officinalis* inflorescences performed by T- student test.

	***p* value**
	OA
treatment	n.s.

Table S33. Content of sterols conjugated in sterol glycosides in *C. officinalis* inflorescences. Data which do not share a common letter are significantly different.

**Compound**	**Content [μg/g DW± SD]**	
	**C**	**CH**
cholesterol	20.35±2.64 a	10.86±1.65 b
campesterol	34.21±2.08 a	22.29±0.67 b
stigmasterol	72.13±6.17 a	46.37±1.69 b
sitosterol	122.69±13.45 a	216.43±16.51 b
**Total sterol glycosides:**	**249.38**	**295.94**

Table S34. Analysis of the chitosan treatment on sterol glycosides content in *C. officinalis* inflorescences performed by T- student test.

	***p* value**			
	cholesterol	campesterol	stigmasterol	sitosterol
treatment	<0.01	0.006	0.01	0.002

Table S35. Content of oleanolic acid (OA) saponins in *C. officinalis* inflorescences. Data which do not share a common letter are significantly different.

Compound	Content [µg/g DW± SD]	
	**C**	**CH**
OA	65472.95±9693.84 a	51055.10±5406.90 a

Table S36. Analysis of the chitosan treatment on oleanolic acid (OA) saponins content in *C. officinalis* inflorescences performed by T- student test.

	***p* value**
	OA
treatment	n.s.

Table S37. Basic physical and chemical charactrerization of universal soil „Athena" including: pH, salinity, concentration of nitrogen (N), potassium oxide (K_2O) and phosphates (P_2O_5).

Parameters	Units	„Athena" soil
pH	-	5,5-6,5
Salinity	g/L	1,5
N	mg/L	180
K_2O	mg/L	220
P_2O_5	mg/L	160
